# Medicinal Animals and Plants as Alternative and Complementary Medicine in Southern Regions of Khyber Pakhtunkhwa, Pakistan

**DOI:** 10.3389/fphar.2021.649046

**Published:** 2021-08-20

**Authors:** Sakina Mussarat, Rehman Ali, Shandana Ali, Ramzi A. Mothana, Riaz Ullah, Muhammad Adnan

**Affiliations:** ^1^ Department of Botanical and Environmental Sciences, Faculty of Biological Sciences, Kohat University of Science and Technology, Kohat, Pakistan; ^2^ Department of Zoology, Faculty of Biological Sciences, Kohat University of Science and Technology, Kohat, Pakistan; ^3^ Department of Pharmacognosy, College of Pharmacy, King Saud University, Riyadh, Saudi Arabia

**Keywords:** zootherapy, ethnobiology, livestock, Khyber Pakhtunkhwa, quantitative indices

## Abstract

**Background:** Local communities use animals and plants as common traditional therapies for various diseases. The study aimed to document animals and animal-plant mixture recipes that are used as alternative and complementary medicine in southern regions of Khyber Pakhtunkhwa, Pakistan.

**Methods:** The data were collected (2017–2018) in three remote areas (Dera Ismail Khan, Bannu, and Lakki Marwat) through questionnaires and face-to-face interviews with local inhabitants. Data on ethnomedicinal uses and cultural values of animal products or parts and their mixture with plants were analyzed using various indices such as frequency of citation (FC), informant consensus (FIC), and fidelity level (FL) to find the highly preferred species in the area.

**Results:** A total of 185 informants (117 females and 68 males) were interviewed. The study documented 32 animal species, vertebrates (*n* = 24) and invertebrates (*n* = 8), for curing 37 types of diseases. Mammals (*n* = 13) were among the most commonly utilized species followed by birds (*n* = 8), arthropods (*n* = 7), reptiles (*n* = 2), and fishes and annelids (*n* = 1 each). Among the reported animals, *Herpestes edwardsi* (mongoose), *Macaca mulatta* (monkey), *Labeo rohita* (rohu), *Oryctolagus cuniculus* (rabbit), and *Streptopelia decaocto* (dove) were the newly reported species used as alternative medicine. The meat of *Capra hircus* (goat), monkey, and rabbit was used to treat chronic diseases such as hepatitis C, cancer, epilepsy, and asthma. A total of 17 plants belonging to 15 botanical families were used in combination with animal parts/products. The commonly used families were Piperaceae (31%) followed by Apiaceae (27%). The notable plant species in combination with animal products were *Curcuma longa, Piper nigrum, Coriandrum sativum, Brassica rapa,* and *Phoenix dactylifera*. Seeds were the highest used part in animal-plant mixture recipes. *Gallus gallus* (chicken) and *Columba livia* (pigeon) secured the highest (FC = 28) and (FL = 80%), respectively. FIC results had shown the highest degree of consensus for general body weakness (FIC = 0.88) and pyrexia (FIC = 0.86).

**Conclusion:** Our findings suggest that local communities in the southern regions of Khyber Pakhtunkhwa have substantial knowledge about the formulation of ethnomedicines from both flora and fauna that need urgent documentation to avoid eroding and for conservational purposes. The newly reported phytozootherapeutic recipes and animal species can potentially be a source of pharmacologically active constituents and should be checked experimentally for further confirmation.

## Introduction

Since ancient times human beings are aware of ethnobiological uses and depend on fauna and flora for their medicines, food, clothing, and other resources of living (Lohani et al., 2008). About 170 member states (88%) of the World Health Organization (WHO) claimed to use complementary and alternative medicines in 2018 (WHO, 2019). Approximately, 65% of the world population relies on alternative medicines derived from natural resources for their primary health care Cragg and Newman (2013) as these are comprised of different bioactive compounds which may be more effective with the least toxicity as compared to synthetic drugs (Oudhia, 1995; Alves and Rosa, 2005). Traditional Chinese medicines, comprising more than 1,500 animal species, Unani medicine consists of about 500 species of invertebrates, while Ayurvedic system of medicine also depends on animal species and their products along with medicinal flora that denotes the importance of fauna and flora in healing ailments (Oudhia, 1995; Tripathy, 1995; Alves and Rosa, 2005).

Pakistan hosts remarkable biodiversity of both fauna and flora Ali (1998), Khan (2004), Mirza and Wasiq, (2007) where most medicines are of plant origin. Tibb-e Islami Dawa Khana (herbal drug markets) reported 600 plant species, general practitioners and tabibs (GPs of Unani medicine) used about 50,000 species of plants, and many unlicensed health practitioners spread in remote hilly and rural areas of Pakistan are using more than 200 plant species in crude drug preparation (Umair et al., 2019). Rural people, who have century’s old traditional knowledge transferred from their ancestors and propagated from generation to generation, play a vital role in disease management. They heavily depend on this knowledge of the traditional system due to poverty, and lack of modern medical facilities, so this information is restricted to rural areas (Sandhya et al., 2006; Ibrar et al., 2007).

The uses of plants as alternative medicine are very common and almost documented both in monoherbal and polyherbal forms in the study area Adnan et al. (2014a), Mussarat et al. (2014), Adnan et al. (2018), Malik et al. (2018), Mussarat et al. (2021) however, only a few reports are available on animal uses from Pakistan (Arshad et al., 2014; Ali et al., 2017; Altaf et al., 2017; Altaf et al., 2018; Shams et al., 2019; Altaf et al., 2020; Ahmad et al., 2021). The documentation of floral and faunal traditional recipes is indispensable before its depletion due to increasing urbanization, modernization, and industrialization. It is essential to report the traditional knowledge regarding plant and animal uses of individual human communities in unexplored regions. Moreover, with the increasing challenge of microorganisms’ resistance against existed allopathic medicines, there is a dire need to check new combinations of natural constituents with additive and synergistic effects. Therefore, the study has been planned with the objectives to document 1) recipes comprised of animals, animal parts, or animal products 2) recipes comprised of both plant and animal parts/products used by the residents of the southern region of Khyber Pakhtunkhwa, Pakistan, and 3) the highly preferred recipes by quantitative indices. This is may be the first time reported study in the selected area to document traditionally used medicinal animal species and animal-plant recipes. The results of the study may be helpful in the conservation of traditional knowledge and identification of new species as a potential source of alternative and complementary medicine.

## Methods

### Study Area

**FIGURE 1:**
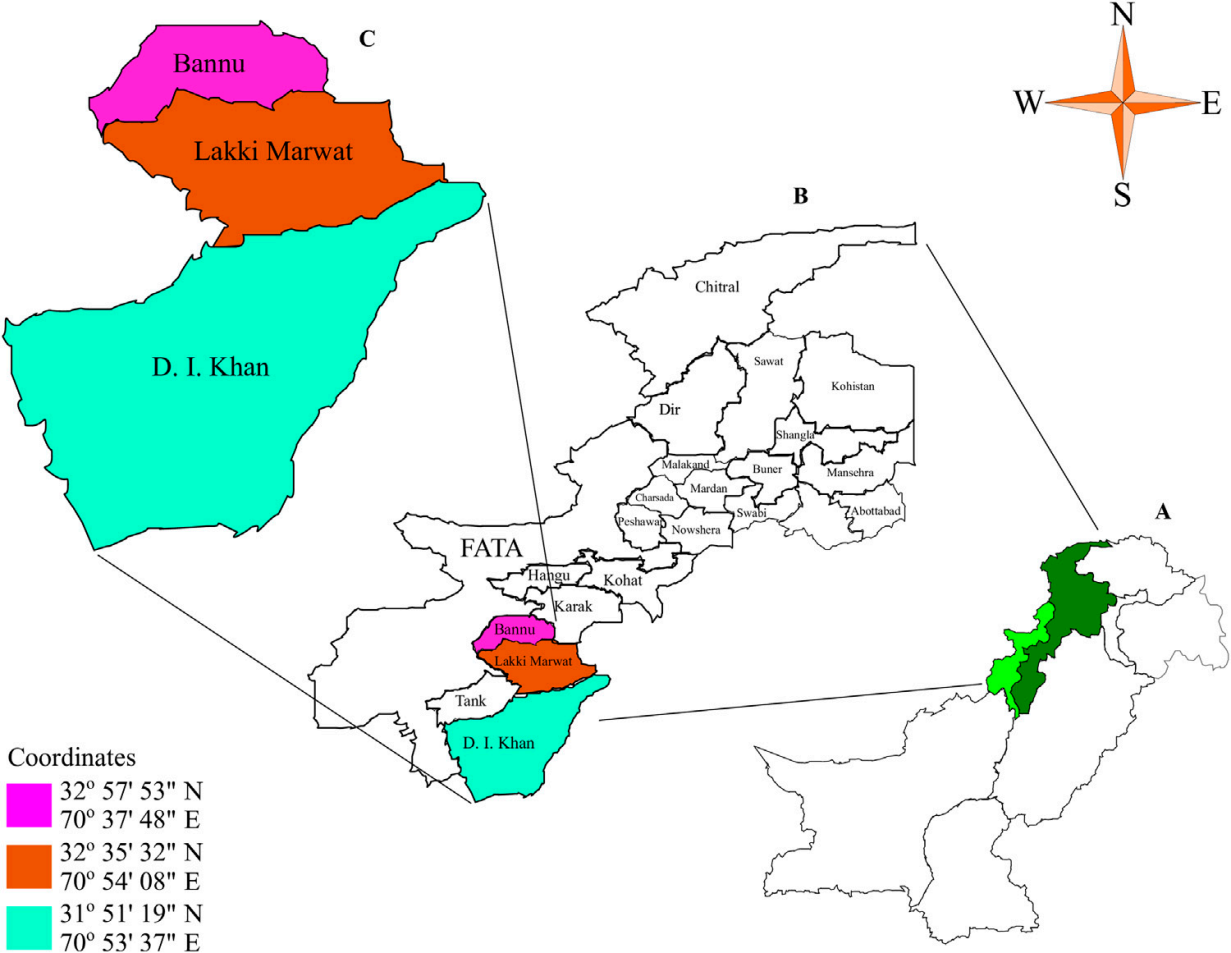
Map of the study area **(A)** Pakistan **(B)** Khyber Pakhtunkhwa **(C)** selectiive districts in southern Khyber Pakhtunkhwa.

This study documents the ethnomedicinal data in three major remote areas (Dera Ismail Khan (D. I. Khan), Bannu, and Lakki Marwat) of Khyber Pakhtunkhwa. D. I. Khan is an area of 7,326 square kilometers and located between 31°15′ and 32°32′ N latitude and between 70°11′ and 71°20′ E longitude (Mussarat et al., 2014). Bannu consists of a total area of 877 square kilometers, with a population of 2,044,074. It lies within the Karakoram mountain ranges between 32°43′ to 33°06′ N latitude and 73°20′ to 70°07′ E longitude. The population is 100% ethnic Pashtun with different casts and different accents i.e. Bannuchi, Wazir, Dawar, Marwat and Bangash. The district forms a basin drained by the Kurram River and Gambila River (Tochi River) which originates from the hills of Waziristan. The Kurram River enters the district from the northwest and from there; it runs towards the south-east, then south into Lakki Marwat (Shaheen et al., 2017). Lakki Marwat is located between 32°161′ N latitude and 70°191′ E longitude at an altitude of 200–1,000 m above sea level (Figure 1). Different ethnic groups living in the study area majority of them were Pashtun with a little difference in their accent. Urdu and English were also spoken and understood by a large number of people. Those living in the urban area possess good socio-economic conditions, having a strong belief in traditional medicine for their primary health care due to the side effects of allopathic medicines. The people living in villages have low-income status and low educational, occupational, and financial level and depend on flora and fauna for their income and primary health needs. These three regions were occupied with a diversity of animals and plants and most of the people of rural as well as urban regions depend upon plants and livestock. Being a rich diversity center, dominant, plant and animal species are *Vachellia nilotica* (L.) P.J.H. Hurter & Mabb *Calotropis procera* (Aiton) W.T. Aiton, *Withania coagulans* (Stocks) Dunal*, Zizyphus,* and cattle, goat, common quail*,* and grey francolin*,* respectively.

### Ethical Approval

This research study was duly approved by the Ethical Research Committee of Kohat University of Science and Technology (KUST) Ref. No. KUST/Ethical Committee/17-12 before the field survey concerning ethnomedicinal data collection and intellectual property rights of local inhabitants. Moreover, ethical guidelines of the International Society of Ethnobiology (http://www.ethnobiology.net) were also followed during the field survey.

### Field Survey and Data Collection

The data collection was carried out from 2017 to 2018. Semi-structured questionnaires were designed to collect ethnomedicinal knowledge of local people about animals and plants. Questionnaires and interviews were conducted to document traditional uses of the animal, animal parts, animal-derived product(s), and animal-plant recipes. Before interviewing, a discussion was held with the informants through the assistance of local elders to elaborate on the objective of the study. This was done to clarify the purpose and build the confidence of the respondents to provide reliable information without any suspicion. Data were collected from 185 local informants including farmers, housewives, teachers, and traditional health practitioners. Prior to data collection, oral, and written informed consent was taken from informants for publication. All informants voluntarily participated in the study. The selected informants were well-known in the community due to their practice of using animals and plants as medicines for their primary health care. These local respondents of the regions were aged between 21 and 79 years. Data were collected in the local language Pashto and Saraiki. There was no need for an interpreter during data collection.

### Animal and Plant Species Identification

Mammals, birds, reptiles, and fish species were photographed and recognized using field guides “Mammals of Pakistan” Roberts (2005a); Roberts (2005b), “Birds of Pakistan” Roberts (1991), “Amphibian and Reptiles of Pakistan” Khan (2006), and “Freshwater Fishes of Pakistan” Mirza (1975). Animal species were thoroughly identified with the help of standard taxonomic keys and available published literature (Day, 1889; Pocock, 1900; Mallon, 1991; Roberts, 1997; Mirza and Wasiq, 2007). Invertebrates and other small animals were captured, identified and after that, they were released again. While large animal species were recognized in the field as per the respective folk description and subsequently with the aid of photo snaps. These photographs were submitted in the Taxonomy Lab of the Department of Zoology, KUST, Kohat. Moreover, the species scientific names were checked and corrected by using the Global Biodiversity Information Facility (https://www.gbif.org) and Catalogue of Life (https://www.catalogueoflife.org).

Plants used in combination with animal species in traditional therapy were collected from the study area and identified at the Department of Botanical and Environmental Sciences, KUST, Kohat and submitted to the herbarium. Voucher numbers were given in the table with each plant species. Plant name correction and their synonym were checked online from “medicinal plant name services-KEW” (http://mpns.kew.org/mpns-portal); “world flora online” (http://www.plantsoftheworldonline.org), and “the plant list” (http://www.theplantlist.org).

### Data Organization

The collected data on ethnomedicinal animals, plants, and ethnography of the respondents were organized into tables and figures. Animal products and parts were categorized into meat, milk, fat, liver, hoofs, bone, spleen, hide, eggs, and the whole animal, etc. Plant parts were categorized into leaves, roots, stem, whole plant, seeds, fruit, and flower, etc.

### Data Quality Assurance

For data verification, each informant was visited at least three times. Only validated and relevant data were subjected to further organization and analysis process. Moreover, authors were trained to collect medicinal plants and animals from the area as well as combination therapy formulation-related information, their uses, doses and concentrations, disease treated, point out missing information, and duplication of materials to maintain data quality.

### Literature Search

**FIGURE 2:**
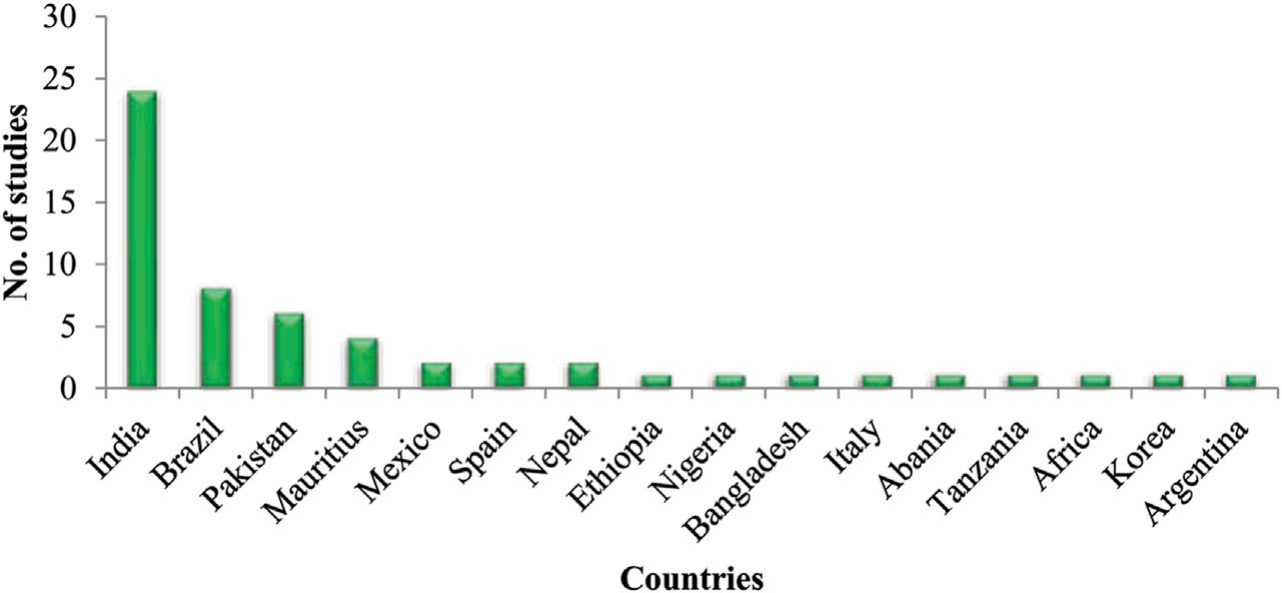
Number of ethnozoological studies conducted throughout the world.

A literature search was performed for retrieving published articles about the traditional use of animals as medicine. Articles published between 1970 and 2021 were retrieved by using different key words like “ethnobiology,” “traditionally used animals,” “ethnozoology”, “animal use as medicine,” and “ethnomedicinal application of animals” in different search engines. Studies that reported the use of animals as medicine were selected and included in this study. Country name and number of studies in each country were counted and a graph comparison was shown **(** Figure 2 **)**.

### Quantitative Analysis

Data on ethnomedicinal uses and cultural values of animal products or parts and their mixture with plants were analyzed using various indices such as frequency of citation (FC), informant consensus (FIC), and fidelity level (FL) to find the highly preferred species in the area statistically.

#### Frequency of Citation (FC)

FC is the number of local respondents who reported ethnomedicinal uses of each animal species (Hoffman and Gallaher, 2007).

#### Informant Consensus (FIC)

FIC on the reported cures of a given group of ailments was calculated as an informant consensus factor. FIC within a community designates the widely used species and thus helps in the selection for phytochemical and pharmacological studies. Reported ailments were grouped into major categories. FIC values are high when one or few animals are reported by many informants to treat a specific ailment, while low FIC values indicate that informants do not agree over which animal to use (Hoffman and Gallaher, 2007).

The FIC can be calculated as; FIC = nur − nt/nur−1.

Where FIC = Informants consensus factor, nur = number of used citations in each category, and nt = number of species used.

#### Fidelity Level (FL)

Fidelity level (FL) helps recognize the most favored species used to treat a special ailment by the respondents (Hoffman and Gallaher, 2007). FL value of highly preferred animals is greater than the value of less preferred animals. FL value is always calculated in terms of the informant’s percentage claiming the use of a definite species for the same ailment. The FL values indicate the importance of certain species for a particular purpose. The reported diseases were grouped into major classes for the calculation of FL values. FL values were calculated as;

FL = Ip/Iu×100. Where Ip indicated the number of informants who reported animal utilization for a specific disease and Iu is the total number of informants who mentioned the same animal for any disease. It is assumed that those medicinal animals which are used frequently by most respondents for the same disease category are more likely to be scientifically effective (Hoffman and Gallaher, 2007).

## Results

### Demographic Profile of Respondents

The local communities of the remote areas have substantial awareness concerning ethnomedicines. During the survey, both male (36.75%) and female (63.24%) respondents of different age groups were questioned (Table 1). The findings depicted that respondents having age >40 years secured a high percentage (*n* = 135, 73%) as compared to respondents <40 years with a percentage of (*n* = 50, 27%). The informants were mostly literate comprising of intermediate, university (24.3% each), and primary level (18.9%) of education. The occupation-wise order of the respondents was: teachers (52.43%), farmers (16.21%), housewives (24.32%), and practitioners (7.02%).

**TABLE 1:** Socio-demographic characteristics of the respondents (*n* = 185) in ethnobiological data collection.

Variables	Total	Percentage (%)
Gender		
Male	68	36.75
Female	117	63.24
Age groups		
21–29	15	8.10
30–39	35	18.91
40–49	50	27.02
50–59	32	17.29
60–69	45	24.32
70–79	8	4.32
Education level		
Illiterate	60	32.43
Primary	35	18.91
College	45	24.32
University	45	24.32
Occupation		
Females		
Housewives	45	24.32
Primary teacher	40	21.62
Secondary teachers	32	17.29
Males		
Farmers	30	16.21
Primary teachers	15	8.10
Secondary teachers	10	5.40
Traditional Practitioner	13	7.02

### Ethnomedicinal Use of Animals

**FIGURE 3:**
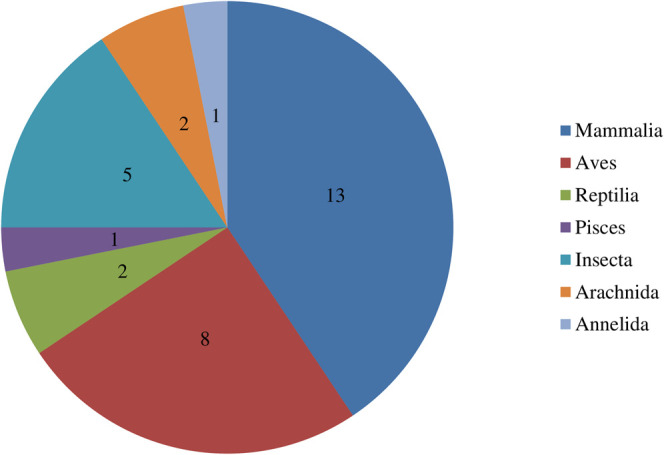
Number of animal classes used in zootherapy practices by local communities.

The present study documented 32 animal species used by the local communities for curing 37 diseases **(** Table 2). These animal species were comprised of both vertebrates (24 species) and invertebrates (8 species). Thirteen species were belonging to class Mammalia, eight to Aves, five to Insecta, two to Arachnida, two to Reptilia, one to Pisces, and one to Annelida (Figure 3). Animals such as *Felis catus, Camelus dromedarius*, *Bos taurus, Equus asinus, Capra hircus, Canis aureus, Paraechinus micropus, Oryctolagus cuniculus, Funambulus palmarum* were used for treating different types of diseases including epilepsy, cancer, hepatitis, night blindness, whooping cough, asthma, and brain hemorrhage among others.

**TABLE 2:** Medicinal uses of animal species and their body parts/products in southern regions of Khyber Pakhtunkhwa (Pakistan).

Animal zoological name/English/Local	Area	Product/body part used	Additives	Recipe and disease treated	Dosage and treatment duration		FC
					Children	Adults	
**Mammalia**							
*Felis catus* Linnaeus, 1758/Cat/Billi	D.I. Khan	Milk	Water	Milk is administered orally for epilepsy	NA	4–5 spoons daily for 4 days	3
*Camelus dromedarius* Linnaeus, 1758/Camel/Oontni/Usha	D.I. Khan	Milk	NA	Milk is administered orally for joint and back pain, to reduce obesity, and cure cancer	NA	One glass daily	13
		Bone of knee	NA	Bone is buried in the earth to avoid termites’ nuisance at home	Not used	Not used	
	Bannu, Lakki Marwat	Milk	NA	Milk is administered orally to cure Hepatitis B and C	One cup per day	Two cups per day	
*Bos taurus* Linnaeus, 1758*/*Cow/Gaae, Ghwa	D.I. Khan	Butter	NA	Butter is prescribed for bone fracture	Once a day	Once a day	22
		Spleen	NA	The cooked spleen is used for drooling in children and to strong teeth	Once a week	Once a week	
		Udder	Water	The udder is prescribed for lactating mothers to increase milk quantity	NA	Twice a week	
		Curd	Eggs	One cup of curd is mixed with 2 eggs and prescribed for hair growth	NA	Once a week	
		Hide	NA	For storage of water	Not used	Not used	
		Blood	NA	Blood of cow, goat, camel, buffalo, and sheep is used to induce fertility and increase the number of fruit on plants	Not used	Not used	
	Lakki Marwat	Liver	Oil	Fried liver is used to treat night blindness	NA	NA	
		Tongue	Oil	The tongue is cooked and used to treat stuttering	For 7 days	NA	
		Omasum	Water	Omasum is cooked and used for digestive problems (pain, gas, and indigestion)	NA	For 7 days regularly	
	Bannu	Milk and butter	NA	Cow milk is mixed with butter and used to relieve gastric pain	Half glass once a day	One glass a day	
*Equus asinus* Linnaeus, 1758*/*Donkey/Kaaligadhi, Khra	D.I. Khan, Lakki Marwat	Urine	Mix any color with it	Urine is used for whooping cough	Half cup twice a week	Half cup twice a week	6
	Bannu	Bones	NA	Donkey bones are crushed to make powder and administered orally to cure epilepsy patients	NA	NA	
	Bannu, D.I. Khan, Lakki Marwat	Milk	NA	Milk is used for whooping cough and asthma	Half glass for a week	One glass at night for 1 week	
*Capra hircus* Linnaeus, 1758*/*Goat/Bakri/Wooza	D.I. Khan, Lakki Marwat	Milk	NA	Fresh milk is administered orally for blisters in the mouth and used topically on the head for dizziness	4–5 spoons	5–6 spoons	15
		Liver	NA	Goat liver is fried in a frypan to obtain the extract for curing night blindness	NA	One drop once a day	
		Skin	NA	The skin is heated up and tied around the arm to heal bone fracture	For 1 week	For 1 week	
		Hide	NA	Goat hide is used to relieve joints pain	Once a day	NA	
		Head meat	NA	The head meat is used for the treatment of hepatitis C	Once a day	NA	
*Homo sapiens* Linnaeus, 1758*/*Human being/Insan	Bannu, D.I. Khan	Milk	NA	Female milk is heated up and used to cure cough, flu, and as a drop in the ear of children to relieve earache	One time for 3 days at night	NA	8
*Canis aureus* Linnaeus, 1758/Jackal/Geedar	D.I. Khan	Skin	NA	Skin in the form of a “hat” is used for brain hemorrhage	NA	1 week	1
*Herpestes edwardsi* E. Geoffroy Saint-Hilaire, 1818/Mongoose/Neola	D.I. Khan	Ankle bone	NA	The bone is tied to the thighs of a pregnant woman to ease delivery. Mongoose is sacrificed and cooked with pumpkin to cure joint pain	NA	One time	1
*Macaca mulatta* Zimmermann, 1780*/*Monkey/Bander, Beezo	Lakki Marwat	Meat	NA	Meat is cooked properly and used for severe cough	One time	One time	1
*Paraechinus micropus* Blyth, 1846/Hedgehog/Kharpusht, Jeggay	Lakki Marwat	Meat	NA	Meat is cooked and used for the treatment of cancer	NA	NA	1
*Oryctolagus cuniculus* Linnaeus, 1758*/*Rabbit/Khargosh/Soya	Bannu	Skin	NA	Wearing of rabbit skin in hand or foot is used to relieve bone pain	NA	One time	4
	Lakki Marwat	Meat	Oil	Meat is used to treat asthma	Once a day	Twice a day	
*Ovis aries* Linnaeus, 1758/Sheep/Bhaer, Mazh	Bannu	Skin	NA	Wearing of sheep skin is used for the treatment of severe cough, fever, and paraplegia	NA	One time	5
	D.I. Khan	Skin	NA	Skin is worn like clothes to treat muscle and joint pain as well as winter fever	For a week	For a week	
	Lakki Marwat	Wool	NA	The sheep wool is burnt in the fire to make ash and used for healing wounds	One time daily	One time daily	
*Funambulus palmarum* Linnaeus, 1758*/*Squirrel/Gulehri, Korhibili	Bannu	Meat	Oil	Fried meat of squirrel in oil is used to treat epilepsy patient and relieve pain	NA	Once a day for 1 week	2
		Tail	Oil	Fried tail of squirrel in oil is used for hair growth	NA	Two times in a week	
	D.I. Khan	Oil/fat	NA	Oil of squirrel is used for the treatment of baldness especially in male	NA	Once a day	
**Aves**							
*Corvus splendens* Vieillot, 1817/Crow/Kaan, Laagra	Bannu, D.I. Khan	Tongue	Salt	The tongue of a crow is fried and eaten for stuttering or crow’s drinking water is given to a child	NA	One time a day for 3–5 days	4
*Anas platyrhynchos* Linnaeus, 1758/Duck/Batakh	D.I. Khan	Meat	Salt and oil	Cooked meat is used for anemia and kidney problems	NA	Once a week	6
		Fat	Salt	Fat is used for relieving piles pain and healing of injuries	NA	NA	
		Liver	Oil	Cooked liver is used to speed up hematopoiesis for curing anemia	NA	NA	
*Gallus gallus* Linnaeus, 1758*/*Chicken/Murghi	Lakki Marwat	Egg	NA	Egg is boiled and whole yolk is removed. The yolk is cooked unless and until the oil come out of it. This oil is used for joints pain, hair fall, and body massage	NA	Two times daily	28
			Milk	Raw egg is mixed with milk. It has great nutritional value and is used for blood pressure	NA	One time	
		Meat	NA	Meat is cooked in water to make soup. It is used for cough, fever, asthma, and weakness	Once a day	Once a day	
		Egg yolk	NA	Egg yolk is separated and applied on the head of children for early tooth eruption	One time daily	NA	
		Fat	NA	Hen fat oil is extracted through cooking. The oil is then used for chest pain and ear pain in children	Two times daily in case of chest pain	NA	
	Bannu	Eggs, meat	Sugar, some spices	Raw eggs are mixed with boiled milk to treat cough. Make a sweet dish of eggs and is used for backache. Soup is used for chest infections. Hen fat is used for tooth eruption	One egg in one cup of milk at night	One egg in one cup of milk at night	
	D.I. Khan	Skin	NA	Skin is used for treatment of blisters and pimples	NA	Once a day	
		Fat	NA	Topically used on gums of children to easy tooth eruption	Twice a day	NA	
		Eggshell	NA	Eggshell is hanged using a piece of thread to avoid lizard nuisance at home and in room, etc.	Not used	Not used	
*Struthio camelus* Linnaeus, 1758/Ostrich/Shutar murgh	D.I. Khan	Fat	NA	Fat is topically applied on hands and feet of children for walking at an early age	Once a daily	NA	1
*Columba livia* J. F. Gmelin, 1789/Pigeon/Kabooter/Kawtara	Bannu	Meat	Salt	Pigeon meat is used for early onset of puberty in young girls	NA	One time at night daily for 1 week	4
	D.I. Khan	Egg	NA	Boiled eggs are used to treat children for stammering	One egg per day for almost 1 week	NA	
*Passer domesticus* Linnaeus, 1758/House sparrow/Chirya, Murghya	Bannu	Droppings	Mother milk	Droppings of sparrows is mixed with mother milk and administered orally to children for the treatment of diarrhea and to relieve abdominal pain	Two spoons twice a day	NA	5
**Reptilia**							
*Saara hardwickii* Gray, 1827*/*Indian spiny-tailed lizard/Sanda	Lakki Marwat	Fat	NA	Fat is converted into oil and applied topically for joints pain, sexual enhancement, body massage, and muscle ache	NA	Two times daily	2
	D.I. Khan	Fat	NA	Sanda oil is used topically for muscle and bone pain	NA	Once time daily	
*Echis carinatus* Schneider, 1801/Snake/Manger	Bannu	Whole animal	NA	Snake is boiled to make soup and is used for the treatment of cancer and all non-curable diseases	NA	Two teaspoons at morning time for 3-days	3
**Pisces**							
*Labeo rohita* Hamilton, 1822*/*Rohu/Machli, Kab	Bannu	Liver	NA	Cod liver oil is used for the treatment of asthma, rickets, joints pain and skin fairness	One time daily	One time daily	11
	D.I. Khan Lakki Marwat	Bones	Honey	Fish skeleton is cooked unless and until it turns black and then ground to make powder of it. A small amount of powder is mixed with one spoon of honey to relieve cough and chest pain	One time daily	Two times daily	
	D.I. Khan	Oil	NA	Oil is topically applied for joint pain	NA	One time per day	
		Gall bladder	NA	The gall bladder is dried up for a long period in a shady place and used for kidney pain and removal of kidney stones	NA	Twice a day	
**Insecta**							
*Ochetellus glaber* Mayr, 1862*/*Ant/Mizhai	Bannu	Mud house	Water	Mud house paste is mixed with water and applied topically for curing mumps	Once a day	Once a day	1
*Apis cerana* Fabricius, 1793/Shehad Makhi/Asian honey bee/Muchya	Bannu, D.I. Khan	Honey	Milk	Honey is mixed with milk and used as an anti-venom	Thrice a day	Thrice a day	21
			Milk	One teaspoon of honey is mixed with two teaspoons of milk and used at night on the face for freshness and fairness	NA	One time daily at night	
*Sceliphron caementarium* Drury, 1773*/*Mud wasp/Girain	D.I. Khan	Mud house	Water	Mud house is mixed with water and used for vomiting	Two times a day	NA	3
*Musca domestica* Linnaeus, 1758*/*Housefly/Mach	Bannu	Whole insect	Chicken soup	Forty houseflies are mixed with chicken soup to treat malaria	Ten houseflies per day	NA	2
	Lakki Marwat	Whole insect	Sugar	Houseflies are mixed with sugar to treat typhoid	NA	One time	
**Arachnida**							
*Orthochirus pallidus* Pocock, 1897/Scorpian/Bichoo, Larham	D.I. Khan	Whole animal	Oil	Scorpion is cooked in oil to make a fine mixture locally called “roghan” and used topically for bell’s palsy and paralysis	Two times a day	Two times a day	9
	Bannu	Whole animal	NA	Scorpion is killed and the poisonous part is removed and used to treat cancer and non-curable diseases	NA	NA	
*Stegodyphus pacificus* Pocock, 1897/Spider/Makri	D.I. Khan	Web	Water	Cobweb is mixed in water and administered orally to treat vomiting in children	Twice a day	NA	2
**Annelida**							
*Hirudinaria manillensis* Lesson*/*Leech/Keera, Jalam	Bannu, D.I. Khan	Whole insect	NA	Human beings used leeches for sucking blood from large pimples and also used for blood purification	NA	One time	8

NA; data not available.

### Ethnomedicinal Use of Animal Products/Parts

**FIGURE 4:**
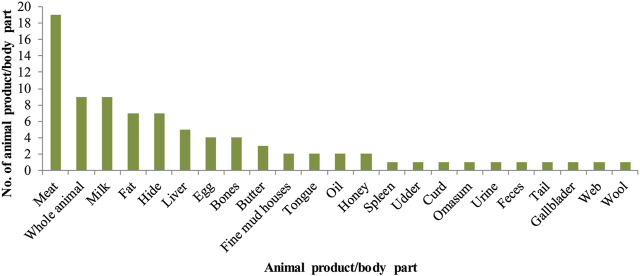
Number of animal products or body parts used in ethnomedicine.

The most widely utilized animal products/parts were meat, used in 19 recipes followed by whole animal and milk, used in nine recipes. While other animal products/parts including fat, oil, hide, liver, eggs, bones, and butter were among the least used products (Figure 4). The meat of different animal species such as goat, rabbit, monkey, and Indian palm squirrel was used for treating diseases like hepatitis C, cancer, epilepsy, asthma, and weakness. Similarly, eggs of chicken and pigeon were utilized to treat blood pressure, fever, cough, joint pain and chest infection. Milk of camel, cattle, cat*,* and the donkey was reported for several ailments and believed to be effective in relieving joints and back pain, gastric pain, treat cancer, paralysis, whooping cough, and reduce obesity. Among the least used products/parts, animals’ fat and oil were practiced to treat baldness, joint and muscle aches, and for sexual enhancement.

### Phytozootherpy Recipes

**FIGURE 5:**
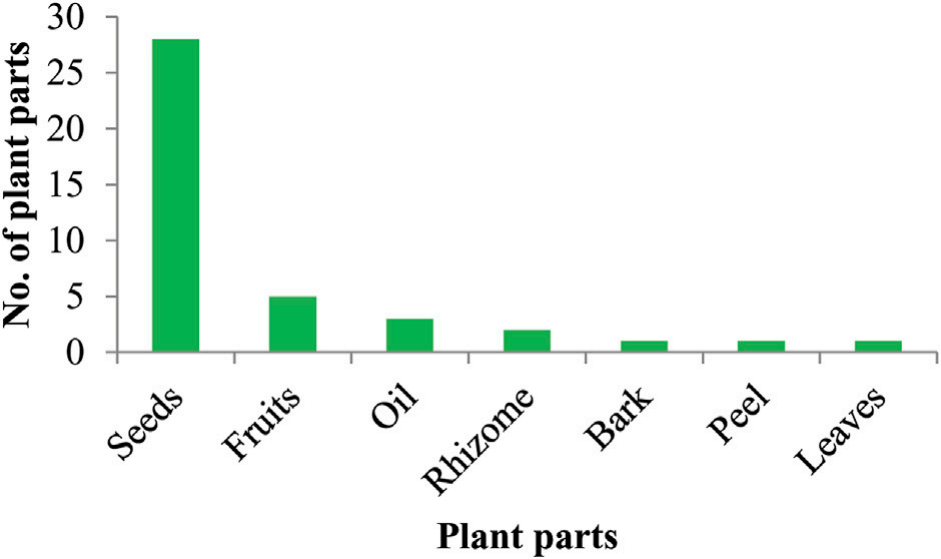
Plant parts used in the preparation of ethnomedicine.

This is the first reported study from the area describing the healing practices of animal-plant mixture recipes. A total of 17 plants belonging to 15 families were used in combination with animal parts/products. The most commonly used botanical family was Piperaceae (31%) followed by Apiaceae (27%), Brassicaceae, Solanaceae, Zingiberaceae and Arecaceae (5%) each. The notable plant species were *Curcuma longa* L.*, Piper nigrum* L.*, Coriandrum sativum* L.*, Brassica rapa* L.*,* and *Phoenix dactylifera* L. (Table 3). Seeds were mostly used plant parts in animal-plant mixture recipes following fruits, oil, and rhizome. While bark, peel, and leaves were the least commonly used plant parts (Figure 5).

**TABLE 3:** Medicinal uses of animal-plant recipes in southern regions of Khyber Pakhtunkhwa (Pakistan).

Zoological name/Local name	Area	Product/body part used	Botanical name/Local name/voucher no./Family	Part used	Additives	Recipe and disease treated	Dosage and treatment duration		FC
**Mammals**									
*Bos taurus* Linnaeus, 1758*/*Cow/Gaae, Ghwa	D.I. Khan	Milk	*Curcuma longa* L./Haldi/KUH-1639/Zingiberaceae	Rhizome	Sugar	Half or one spoon of turmeric is mixed in a cup of milk and administered orally to reduce pain in injury	Once a day	Once a day	47
			*Phoenix dactylifera* L./Chwara/KUH-359a/Arecaceae	Fruit	Sugar	Soak four dates overnight in a cup of boiled milk, add some sugar, and are used early in the morning to conceive pregnancy	NA	One cup on 2nd day of menses and use (one cup) for 3 days onwards. Repeat the same for each menstrual cycle for 3 months	
			*Cicer arietinum* L./Chana/KUH-323a/Fabaceae	Seeds	Water	Make a paste of *Cicer arietinum* seeds powder (one spoon), one spoon of lemon juice and milk is mixed and applied topically for pimples	NA	Daily at night for 1 week	
			*Citrus limon* (L.) Osbeck/KUH-327a/Niboo/Rutaceae	Fruit					
		Curd	*Mentha piperita* L./Podina/KUH-353a/Lamiaceae	Leaves	Salt	Crushed fresh leaves of *Mentha piperita*, seeds of *Piper nigrum*, and some salt is mixed in curd for treating stomach problems	2–3 spoons after a meal	5–6 spoons after a meal	
			*Piper nigrum* L./Kali mirch/KUH-462/Piperaceae	Seeds					
		Butter	*Peganum harmala* L./Harmal/KUH-358a/Nitrariaceae	Seeds	NA	Paste of harmal plant and butter is prepared and used topically for an abscess on the body	One time a day	One time a day	
	Bannu, D.I. Khan	Leg bones (Trotters)	*Piper nigrum* L./Kali Mirch/KUH-462/Piperaceae	Seeds	Salt, water	Cow’s leg bone is boiled, add some spices, to make soup and is used to relieve backache and leg pain. It also used as a delicious and nutritious food	NA	One cup per day	
			*Coriandrum sativum* L./Dhania/KUH- 331a/Apiaceae	Seeds					
	Bannu	Milk	*Phoenix dactylifera* L./Kajoor/KUH-359a/Arecaceae	Fruit	Sugar	Cow’s milk is mixed with four dates and prescribed for the treatment of cough and opening of heart valves	One cup daily at night for 1 week	One cup daily at night for 3–4 days	
			*Ricinus communis* L./Castor oil/KUH-449a/Euphorbiaceae	Oil	Egg	Two eggs, four spoons of Castrol oil are mixed with cow milk and is used at the time of delivery to relieve pain	NA	One glass	
		Butter	*Punica granatum* L./Anar/KUH-448a/Lythraceae	Peel	Milk	Powder of dry peel of one Pomegranate is mixed with one jug of milk and two spoons of butter are used for the appendix and gastric pain	NA	One cup before breakfast and at night for 2 days	
*Capra hircus* Linnaeus, 1758/Goat/Bakri, Wooza	Bannu, D.I. Khan	Meat	*Piper nigrum* L./Kali mirch/KUH-462/*Piperaceae*	Seeds	Salt, water	Boiled meat, a teaspoon of black pepper, one teaspoon of garam masala, salt are cooked to make soup and is used for the treatment of Hepatitis B	One cup per day daily for 15 days	One cup daily for 10 days	8
			*Coriandrum sativum* L./Dhania/KUH- 331a/Apiaceae	Seeds					
		Liver	*Coriandrum sativum* L./Dhania/KUH- 331a/Apiaceae	Seeds	Salt, water	The liver is cooked with some spices and used for the treatment of anemia	3–4 pieces daily for almost 7–8 days	3–4 pieces daily for almost 7–8 days	
			*Piper nigrum* L./Kali mirch/KUH-462/Piperaceae	Seeds					
*Homo sapiens* Linnaeus, 1758/Human being/Insan	Lakki Marwat*,* D.I. Khan	Hairs	*Brassica rapa* L./Sarsoon/KUH-428a/Brassicaceae	Oil	Egg	Hairs are dipped into a mixture of Mustard oil and egg for topical use to relieve joints pain and muscle ache	NA	Twice a day	5
*Oryctolagus cuniculus* Linnaeus, 1758/Rabbit/Khargosh	Bannu	Meat	*Solanum lycopersicum* L./Tmatar/KUH-469/Solanaceae	Fruit	Salt, water	Cooked meat is used for the treatment of bronchial diseases	Half cup of soup per day for 4 days	One cup for almost 2 days	5
			*Capsicum annuum* L./Sabzmirch/KUH-468/Solanaceae	Fruit					
	D.I. Khan	Meat	*Piper nigrum* L./Kali mirch/KUH-462/Piperaceae	Seeds	Salt, water	Meat is cooked with some spices and salt for treating Bell’s palsy and asthma	NA	One cup for 3 days	
			*Coriandrum sativum* L./Dhania/KUH- 331a/Apiaceae	Seeds					
*Ovis aries* Linnaeus, 1758*/*Sheep/Bhaer, Mazh	Bannu	Liver	*Piper nigrum* L./Kali mirch/KUH-462/Piperaceae	Seeds	Oil, salt, water	The liver is cooked with spices and used to increase the blood level	Once a day for a week	Once a day for a week	5
			*Coriandrum sativum* L./Dhania/KUH- 331a/Apiaceae	Seeds					
**Aves**									
*Gallus gallus* Linnaeus, 1758/Chicken/Chote chooze	D.I. Khan	Meat	*Piper nigrum* L./Kali mirch/KUH-462/Piperaceae	Seeds	Salt, water	Soup is used for the anemic condition after large injuries and accidents	Once a day for a week	Once a day for a week	8
	Lakki Marwat		*Coriandrum sativum* L./Dhania/KUH- 331a/Apiaceae	Seeds		Soup is used for the chest infection, cough, fever, asthma, and weakness	One time a day	One time a day	3
*Streptopelia decaocto* Frivaldszky, 1838/Dove/Faakhta	Bannu	Meat	*Piper nigrum* L./Kali mirch/KUH-462/Piperaceae	Seeds	Salt, water	Soup of dove meat, add some spices and black pepper is prescribed for the treatment of cough and early onset of puberty in girls	One cup at night for cough for 3 days	One cup daily for 1 month	6
*Columba livia* J. F. Gmelin, 1789*/*Pigeon/Kabooter, Kawtara	Bannu	Meat	*Piper nigrum* L./Kali mirch/KUH-462/Piperaceae	Seeds	Salt, water	Boiled meat of pigeon by adding some spices is used for the treatment of measles and Bell’s palsy	Two times per day for 3-days	3-times per day for almost 1 week	16
			*Coriandrum sativum* L./Dhania/KUH- 331a/Apiaceae	Seeds					
	Lakki Marwat, D. I. Khan	Meat	*Piper nigrum* L./Kali mirch/KUH-462/Piperaceae	Seeds	Salt, water	Soup of pigeons is very effective in cough, asthma, and paralysis	One time daily	One time daily	
			*Coriandrum sativum* L. Dhania/KUH- 331a/Apiaceae	Seeds					
*Passer domesticus* Linnaeus, 1758*/*House sparrow/Chirya, Murghya	D.I. Khan	Meat	*Piper nigrum* L./Kali mirch/KUH-462/Piperaceae	Seeds	Salt, water	The meat of sparrow is boiled by adding some spices is used for the treatment of measles and heart problems	One cup for almost 3-days	As needed	3
			*Coriandrum sativum* L./Dhania/KUH- 331a/Apiaceae	Seeds					
	Lakki Marwat	Blood	Vicia lens (L.) Coss. and Germ. Masoor Daal/KUH-467/Leguminosae	Seeds	NA	Ground pulses (powder) is mixed with blood to make pills and are used for sexual enhancement	NA	NA	
*Acridotheres tristis* Linnaeus, 1758*/*Myna/Maina, Myna	Bannu	Whole animal	*Piper nigrum* L./Kali mirch/KUH-462/Piperaceae	Seeds	Oil, salt, water	Boiled meat of starling by adding some spices and is prescribed to those children who have a problem in speaking and walking	Half cup per day for 2 days	NA	3
			*Coriandrum sativum* L./Dhania/KUH- 331a/Apiaceae	Seeds					
**Insecta**									
*Neoponera venerae* Forel, 1922/Ant/Papeeli	D. I. Khan	Whole body	*Olea europaea* L./Zaitoon/KUH-466/Oleaceae	Oil	Water	Whole ant is cooked in olive oil by adding some water and is used as a drop in the ear to treat deafness	NA	NA	2
*Apis cerana* Fabricius, 1793/Asian honey bee/Shehad ki Makhi/Muchya	Bannu	Honey	*Camellia sinensis* (L.) Kuntze KUH-464/Sabzchay/Theaceae	Leaves	Milk	A spoon of honey is mixed with half spoon turmeric and one glass of milk is used for the treatment of cough and throat infection. Honey is also mixed with green tea to lose extra fat	One cup at night for 1 week	One cup at night for 3-days and Green tea can be used daily	16
			*Curcuma longa* L./Haldi/KUH-436a/Zingiberaceae	Rhizome					
	D.I. Khan	Honey	*Cinnamomum verum* J.Presl/KUH-463/Dar cheeni/Lauraceae	Bark	NA	Powder of cinnamon plant is mixed with honey to treat fever and cough	Twice a day	Thrice a day	
**Arachnida**									
*Orthochirus pallidus* Pocock, 1897/Scorpion/Bichoo, Larham	Bannu, Lakki, D.I. Khan	Whole animal	*Brassica rapa* L./Sarsoon/KUH-428a/Brassicaceae	Seeds	NA	Scorpion is fried in *Brassica* seeds oil and used topically to reduce pain and swelling of a venomous bite	Twice a day	Twice a day	4

NA; data not available.

### Modes of Preparation and Application

**FIGURE 6:**
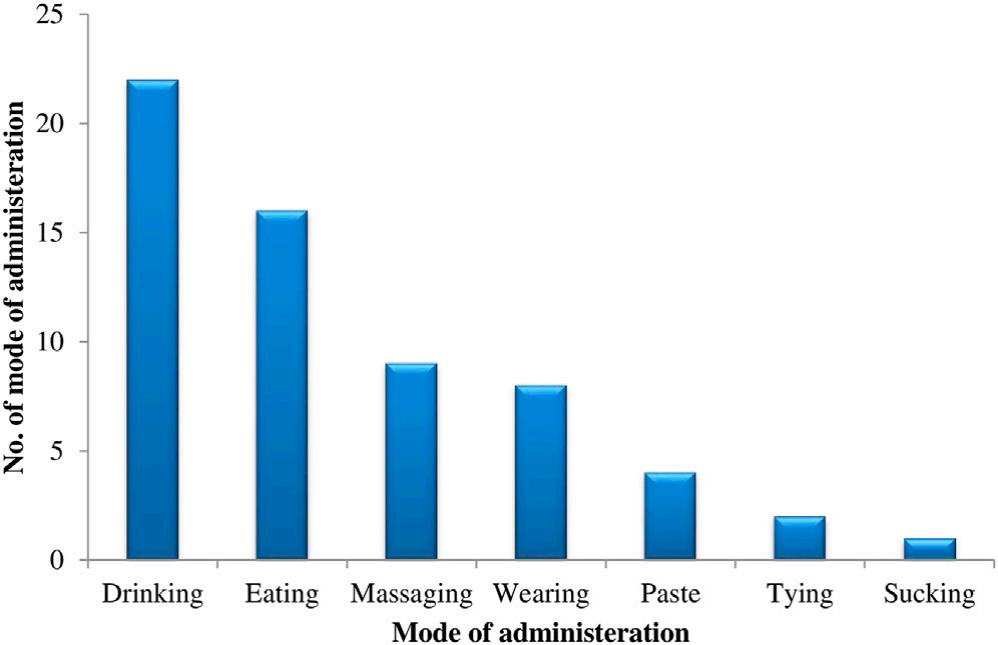
Modes of administration of ethnomedicine.

Mostly practiced mode of administration was drinking used in 22 recipes followed by eating, massaging, and wearing used in 16, 9, and 8 recipes, respectively. Other modes like tying and sucking contributed in less than three recipes **(** Figure 6 **)**. Some of the traditional medicine (liquids and solids) was taken orally, while others were applied topically like, animal hides were worn to treat fever and bones were tied for healing purposes. In the recipes’ preparation, additives like water, honey, oil, milk, sugar, salt, and colors were also used (Table 3).

### Quantitative Analysis

Animal species that were used by the maximum number of respondents to treat various diseases secured a high FC value. In the current findings, the FC values for using animal-based recipes ranged from 1 to 28, while for animal-plant recipes they ranged from 2 to 47. In animal-based recipes chicken (FC = 28), cattle (FC = 22), and Asian honey bee (FC = 21) were the most commonly used species. While ants, ostrich, and jackal were with the lowest (FC = 1 each). Similarly, in animal-plant recipes cattle (FC = 47), Asian honey bee (FC = 16), and pigeon (FC = 16) were with the highest reported FC. FIC results had shown the highest degree of consensus for general body weakness (FIC = 0.88), pyrexia (FIC = 0.86), arthritis (FIC = 0.83), and dermatological diseases (FIC = 0.80) (Table 4).

**TABLE 4:** Informant consensus factor for animal and animal-plant recipes.

Disease categories	Nur	Nt	FIC
General body weakness	34	5	0.88
Pyrexia	22	4	0.86
Arthritis	31	6	0.83
Dermatological	53	11	0.80
Respiratory	39	9	0.79
Gastrointestinal	28	8	0.74
Nervous	25	8	0.71
Reproductive	18	9	0.52
Wounds	11	4	0.7
Anemia	16	7	0.6

Fidelity level was calculated to recognize animal species that were most frequently and preferably used by the local population for curing certain ailments. The fidelity level ranged from 1 to 100%. Animal species with the highest medicinal use in a particular area had a maximum fidelity level. Three animal species, pigeon (FL = 80%), rabbit (FL = 66.6%), and Asian honey bee (FL = 45.94%) scored the highest, whereas goat scored the lowest (FL = 13%) (Table 5).

**TABLE 5:** Fidelity level of highly utilized animal species in traditional therapies.

Animal species	Disease category	Ip	Iu	FL %
*Columba livia*	Nervous	16	20	80.0
*Oryctolagus cuniculus*	Respiratory	6	9	66.6
*Apis cerana*	Dermatological	17	37	46.0
*Labeo rohita*	Dermatological	3	11	27.3
*Gallus gallus*	Pyrexia	8	36	22.2
*Bos taurus*	Gastrointestinal	13	69	19.0
*Capra hircus*	Arthritis	3	23	13.0

## Discussion

Complementary and alternative medicine remained the basic source of primary health care at the doorstep of both urban and rural people. A large population of the world in developing as well as developed countries still depends upon ethnomedicine, despite a great advancement in modern health facilities and allopathic medicine.

The local population of the study area is mostly dependent on ethnomedicine as the first choice of treatment. No previous study has been reported to document traditionally used animal-based and animal-plant recipes from southern areas of Khyber Pakhtunkhwa. Therefore, this study was aimed to fulfill the knowledge gap of reporting ethnomedicine used in southern regions of Khyber Pakhtunkhwa with the main objectives to document 1) recipes comprised of animals, animal parts, or animal products 2) recipes comprised of both plant and animal parts/products and 3) the highly preferred recipes by quantitative indices.

People living in rural areas mostly have a strong belief and prefer traditional medicine (Adhikari et al., 2020). All the informants were having substantial knowledge of using ethnomedicines. Female respondents were found to be more familiar with the use of ethnomedicines than males. Mostly, females were involved in using ethnomedicine due to taking care of their children and families. Most of them were educated and had a strong belief in using ethnomedicine rather than allopathic medicine. The main reason, which female respondents stressed, was the associated side effects of allopathic medicine. The result was supported by earlier reports Mahomoodally et al. (2019) confirming females as the most predominant users of animal-based recipes. However, male respondents were more dominant and actively involved in using ethnomedicines in Nepal, India, and Ethiopia (Borah and Prasad, 2017; Kendie et al., 2018; Adhikari et al., 2020). The ratio of male to female respondents that were interviewed in the present study and those reported earlier may justify this difference.

The participants of age group above 40 years secured a high percentage and were predominantly involved in using ethnomedicines. A similar trend was observed in previous studies conducted in Nepal Adhikari et al. (2020), Ethiopia Kendie et al. (2018), India Borah and Prasad (2017), and Pakistan Altaf et al. (2018) indicating that elder respondents possessed more knowledge and experience regarding ethnomedicines. Mostly respondents stated that they acquired knowledge from their elders. This further strengthens the belief that transmission of folk medicine occurs from elder to younger individuals. In contrast, the possible reason for less ethnomedicinal awareness among youngers could be due to their intention towards urbanization and the least interest in ethnomedicine (Borah and Prasad, 2017). The informants were mostly literate and least being with a primary level of education. However, the illiteracy was also of considerable level due to poor socioeconomic conditions, lack of access to education, and most had opted to farm as their profession because of their elders. Mostly, respondents (16.21%) were farmers having broad knowledge of ethnomedicine.

Thirty-two animal species were reported to be used by the local communities for curing 37 types of diseases including epilepsy, cancer, hepatitis, night blindness, whooping cough, asthma, paralysis, and brain hemorrhage among others. Use of *Camelus dromedarius* for treating cancer, hepatitis, diabetes, *Capra hircus* for enhancing sexual power and asthma, *Oryctolagus cuniculus* for paralysis, and *Ovis aries* for cough, flu, skin burn, weakness and joint pain has been reported earlier and were overlapping the current findings (Altaf et al., 2018). In Bahawalpur-Pakistan, the products/parts of the above-mentioned mammals have also been mentioned for skin diseases, jaundice, rickets, tuberculosis, weakness, paralysis, and asthma (Ahmad et al., 2021).

Similarly, 44 animal species, including *Herpestes edwardsii, Sciurus caroliniensis, Bos indicus, Bubalus bubalis,* and *Vulpes bengalensis* etc., were reported in Assam India to treat 40 diseases. The commonly treated diseases were cancer, asthma, rabies, epilepsy, piles, paralysis (Borah and Prasad, 2017). Local people of Ethiopia and Mauritius used 51 and 32 animal species to treat over 36 and 38 kinds of ailments, respectively (Kendie et al., 2018). Apart from Asia and Africa, animal use in ethnomedicine has also been documented in European countries (Quave et al., 2010; Benitez, 2011). Different active agents that are being used as potential drugs have been isolated from various animals Daly et al. (2005), Cragg and Newman (2013), Rodrguez et al. (2017), Zhan et al. (2020) which show the medicinal importance of the animals. For instance, *Bufo gargarizan,* a traditionally used medicinal animal, is a rich source of bioactive agents and approximately 118 bufadienolide monomers and 11 indole alkaloids have been isolated from it to date. The extracts and isolated compounds exhibit a wide range of *in vitro* and *in vivo* pharmacological effects such as detoxification, reduce swelling, anti-inflammation, antitumor, and immunomodulation (Zhan et al., 2020).

In our findings, mammals remained as the highly utilized animal group for therapeutic purposes. Similar studies have been reported in Pakistan, North-Western Ethiopia, Nepal, and South Africa Whiting et al. (2013), Altaf et al. (2018), Adhikari et al. (2020) where mammals were mostly used as medicines. Most of these animals were domesticated and therefore, easily accessible. The local inhabitants also believed that mammals are the rich sources of proteins, supplementary foods, and medicines and are commonly practiced by the local communities for meat and milk. In contrast, extensive use of insects has also been documented in India (Chakravorty et al., 2011; Borah and Prasad, 2017). However, in some reports reptiles are among the highest animals used in ethnomedicines (Altaf et al., 2020; da Nóbrega Alves et al., 2008; Fernandes-Ferreira et al., 2013). The number of different animal species shows the diversity in an area however, the composition of fauna, accessibility, and availability directly influences the zootherapeutic resources used in any given region (Alves and Rosa, 2007). Different indigenous tribal groups also sacrifice animals for different rituals and religious purposes in keeping with their mythological myths and beliefs. For example, people wear tiger and bear claws around their necks to protect from evils while animals like goats, buffalo, pigeons were sacrificed to please Gods for healing purposes (Solanki and Chutia, 2004; Mahawar and Jaroli, 2006; Alves, 2009).

Meat, whole animal, and milk were among the widely used animal products. Meat as the highest used part of ethnomedicine in Lahore and Jhelum has already been reported (Altaf et al., 2018). Similarly, another study in Ethiopia documented meat and fat as the commonly utilized medicinal products of animals (Kendie et al., 2018). In contrast, in some reports, the highest use of whole animals for medicinal purposes has also been documented (Borah and Prasad, 2017). *Capra hircus, Macaca mulatta, Paraechinus micropus, Oryctolagus cuniculus, Funambulus palmarum, Anas platyrhynchos,* and *Gallus gallus* meat was used to treat hepatitis, cough, cancer, asthma, epilepsy, anemia, weakness, and kidney problems. Similar uses of different animals’ meat have been reported to treat patients suffering from paralysis, asthma, rheumatism, arthritis, poor vision, and tuberculosis (Adhikari et al., 2020). *Columba livia* meat was utilized for early onset of puberty in females in our study. However, patients with paralysis were given cooked meat of *C. livia* in Nepal Adhikari et al. (2020), while fresh blood was used in India for curing the same disease (Mahawar and Jaroli, 2007). Meat and meat products are significant sources of proteins, vitamins (vitamin B12), minerals (zinc, iron, and phosphorus), and provide other essential components to our diet (Biesalski, 2005; Williams, 2007; McAfee et al., 2010). Vitamin B12 (also known as cyanocobalamin) has been found to inhibit the HCV internal ribosome entry site (IRES)-dependent translation of a receptor gene *in vitro* (Lott et al., 2001). Similarly, iron deficiency can lead to severe anemia and is essential for heme and hemoglobin formation (Camaschella et al., 2016). These health improving effects and rich nutritious value could justify the use of meat of different animal species for treating different types of diseases.

The eggs of pigeon and chicken were utilized to treat blood pressure, fever, cough, joints pain, and chest infection. Eggs have been previously reported for treating similar diseases (Altaf et al., 2018). Eggs of *G. gallus* are being used for curing weakness and cough Ahmad et al. (2021), nasal congestion to stop bleeding and dysentery Shams et al. (2019), and reinforces potency and enhances libido (Lev, 2003). Eggshell has been found useful in the treatment of urinary stones (Ahmad et al., 2021). Similarly, the egg shell of *Struthio camelus* has been utilized for treating eye diseases (Lev, 2003). Research shows that eggs have high nutritional value and provide an excellent source of both water-soluble (B1 and B12) and lipid-soluble (A, D, E, and K) vitamins, omega-3-fatty acids, and minerals such as phosphorus and iron (Anton et al., 2006). Moreover, lysozymes from egg albumin have antibacterial, antiviral activities (viral bronchitis, herpetic lesions, stomatitis, etc.), while ova albumin extracted from eggs is effective in preventing hypertension (Dedonder and Morias, 1974; Fujita et al., 1995).

Fats and oil of animals were used to treat baldness, joint, and muscle aches, and for sexual enhancement. It has been proven that fats and oil of animal origin contain omega-3 fatty acids which reduce inflammation (Wilson, 2015). Some studies have also verified that fats/oil of animals is beneficial in neurological disorders, atherosclerosis, thrombotic, and aging effects (Haag, 2003). However, rich fats comprised of a high fraction of saturated fatty acids are considered to be major factors of obesity, body mass gain, and heart disease (Anton et al., 2006). Therefore, the use of animal fat/oil could have adverse effects and may cause potential health issues.

Milk was utilized to relieve joints and back pain, gastric pain, treat cancer, paralysis, whooping cough, and reduce obesity. The findings are in parity with a study conducted by (Altaf et al., 2018). Milk contains nutritious and effective components that reduce joint pain, strengthen the body, and increase sexual potency (Alabdulkarim, 2012; Sabahelkhier et al., 2012). Milk is not only the source of bioactive components, but may also serve as an important delivery medium (Ebringer et al., 2008).

Apart from using animal products, people were also using animal-plant recipes. Plants and plant-derived products are usually used in combination with animal parts and byproducts to enhance the therapeutic effects of ethnomedicines (Borah and Prasad, 2017). Among the reported plant families, the most commonly used were Piperaceae and Apiaceae. Traditionally, individual plants of these families have also been used as folk medicine. In the present study, most of the plants of Piperaceae and Apiaceae were used as spices in recipe formulation as additional ingredients for good taste. Another main reason behind the wide use of these families in folk medicine is having vast pharmacological activities. Different compounds and extracts isolated from plant species belonging to Piperaceae have been experimentally tested for analgesic, antibacterial, antimalarial, and other pharmacological activities (Roersch, 2010). Similarly, plant species of Apiaceae possess antioxidant, anti-diuretic and anti-inflammatory activities (Dehpour et al., 2009; Matasyoh et al., 2009; Miguel et al., 2010; Hajhashemi et al., 2011). Cow milk was mixed with rhizome powder of *C. longa* to reduce injury pain and with honey to treat cough and throat infections. These preventive effects may be due to the presence of several nutrients and bioactive components in milk, among them caseins and whey proteins are of particular importance and have significant roles in immune and digestive systems, reduce blood pressure, antimicrobial, and anticarcinogenic effects (Ebringer et al., 2008). Compounds like curcumin (from *C. longa*) possess anti-inflammatory, antioxidant properties, and having a protective effect for rheumatism, sinusitis, biliary disorders, anorexia and immune diseases (Araujo and Leon, 2001; Yang et al., 2019). Literature has reported, the anti-arthritic effects of curcumin in humans with osteoarthritis (OA) and rheumatoid arthritis (RA) (Hewlings and Kalman, 2017). In the southern regions of Khyber Pakhtunkhwa, *C. longa* has also been used for pain and healing purposes (Adnan et al., 2014b). Similarly, the juice (leaves and barks) of *Alstonia scholaris* in combination with milk was used to treat chronic dysentery Borah and Prasad (2017) while cow urine is mixed with crushed seed of *Sesbania grandiflora* (L.) Pers (Bokful) for the treatment of epilepsy (Borah and Prasad, 2017). Some medicinal preparation where both plant and animals are utilized in combination is also reported from Brazil (Alves et al., 2013; Costa-Neto, 1999; da Nóbrega; Alves and Filho, 2007). However, the role of each component present in most of the mixtures is unclear. In animal-plant recipes, one component may act as a carrier, to enhance the therapeutic activity of each other, or reduce/mask the adverse effect(s) of one another. Hence, the role (s) of different components in mixture recipes should be explored in future research. These traditional ways of different combinations showing antagonistic/synergistic effects and providing new insights in the field of pharmacology that must be checked through *in vitro* and *in vivo* screening.

Cooking, boiling, juice, powder, frying, paste, and smoke were the common modes of ethnomedicine preparation. While drinking, eating, massaging, and wearing were the highly practiced modes of administration. Mostly, animal-based parts and products such as meat are properly cooked before consumption because raw consumption of animals’ parts is haram (forbidden) in the Islamic religion. A similar trend was observed in Nepal where 12 types of preparations were used and cooked meat and other animal parts were commonly practiced (31%) (Adhikari et al., 2020). On the other hand, raw consumption of animals and animal’s parts was the highly used way of taking animals in India and Korea (Kim and Song, 2013; Vijayakumar et al., 2015; Borah and Prasad, 2017). However, the consumption of raw meat may increase the risks of transmitting different types of parasites and diseases to humans (Lohani, 2012; Kim and Song, 2013). Zoonotic diseases can be transmitted by direct contact with animals and also by using animal products as foods and medicines. For instance, raw consumption of milk has been associated with toxoplasmosis infection in the past and such practice is discouraged (Sacks et al., 1982). Therefore, proper preparation methods should be adopted for consuming animals and animals products as ethnomedicines.

Liquid and solids were frequently administered orally, while some were applied topically. These results were following the study conducted by Borah and Prasad (2017) showed that traditional medicines are mostly administered orally as compared to applied topically. However, the dermal application of ethnomedicines is still very reliable in joints pain, muscle aches, wounds, piles and bone fractures (Jaroli et al., 2010; Kim and Song, 2013). Additive substances like water, honey, oil, milk, sugar, salt, and colors were also used in ethnomedicine preparations same as in a previous study by (Kendie et al., 2018). These additives are useful for easy ingestion, increase solubility, decrease/minimize any bitter taste of ethnomedicine.

Comparative analysis showed a limited number of ethnozoological studies conducted across the world. Medicinal use of animals is not extensively reported. India contributed predominantly to the ethnozoological studies and reported a higher number of studies as compared to other countries. Five studies were found to be published in Pakistan from Punjab regions describing the medicinal value of fauna and only one study was reported from Sawabi district of Khyber Pakhtunkhwa. Moreover, few studies were reported in the world where a mixture of plant and animal products was used for medicinal purposes. In this regard, the present data has potential value to the field of ethnobiology. Moreover, most studies were providing insufficient data regarding the traditional use of an animal as medicine. A detailed formulation of traditional medicine should be ensured in the future consisting of 1) accurate name of the reported species (scientific, vernacular, and English) 2) part and product used 3) mode of preparation 4) mode of administration 5) proper dose and number of doses per day 6) duration of treatment and 7) toxicity or adverse side effects (if any).

The quantitative analysis is of great importance in ethnomedicinal studies, because it provides baseline information for further ethnopharmacological studies. Animal species, both in animal-based and animal-plant recipes, having high FIC, and FL revealed the ethnomedicinal importance of these species and are recommended for further ethnopharmacological validation to better understand their zootherapeutic potential and explore the unknown bioactive compounds owing to ethnomedicinal efficacy.

## Conclusion

Our findings suggest that local communities in the southern regions of Khyber Pakhtunkhwa have substantial knowledge about the formulation of ethnomedicine from both fauna and flora. This is the first attempt to document animal-based and animal-plant recipes from southern regions of Khyber Pakhtunkhwa, Pakistan. This study conserves the ethnobiological data and provides bases for pharmacological, phytochemical and synergistic studies. People either use an animal product/part directly or in combination with a plant part for curing different types of human ailments. The findings also suggest that ethnomedicine has a key role in the primary health care system of the study area by using a mixture of both fauna and flora. The animal-plant recipes provide new insights into drug development that could lead to the discovery of novel and effective drugs through synergistic mechanisms. Researchers are especially invited to conduct more research in this field to preserve and document traditional knowledge because this is eroding rapidly due to the death of elder traditional healers/herbalists and the modernization of the upcoming generation. Furthermore, animal products/parts with the highest FL, and FIC should further be studied in the future to assess the pharmacologically active compounds by using *in vitro*/*in vivo* assays.

## Data Availability

The original contributions presented in the study are included in the article/Supplementary Material, further inquiries can be directed to the corresponding authors.
